# A genome-wide scan study identifies a single nucleotide substitution in the tyrosinase gene associated with white coat colour in a red deer (*Cervus elaphus*) population

**DOI:** 10.1186/s12863-020-0814-0

**Published:** 2020-02-10

**Authors:** Gerald Reiner, Kirsten Tramberend, Florian Nietfeld, Klaus Volmer, Christine Wurmser, Ruedi Fries, Hermann Willems

**Affiliations:** 1 Department for Veterinary Clinical Science, Justus-Liebig-University, Frankfurter Strasse 112, D-35392 Giessen, Germany; 2 Arbeitskreis Wildbiologie e.V, Justus-Liebig-University Giessen, D-35392 Giessen, Germany; 3 Department of Animal Breeding, Technical University of Munich, Liesel-Beckmann-Strasse 1, D-85354 Freising-Weihenstephan, Germany

**Keywords:** Red deer, White coat colour, Next generation sequencing

## Abstract

**Background:**

Red deer with very pale coat colour are observed sporadically. In the red deer (*Cervus elaphus*) population of Reinhardswald in Germany, about 5% of animals have a white coat colour that is not associated with albinism. In order to facilitate the conservation of the animals, it should be determined whether and to what extent brown animals carry the white gene. For this purpose, samples of one white hind and her brown calf were available for whole genome sequencing to identify the single nucleotide polymorphism(s) responsible for the white phenotype. Subsequently, samples from 194 brown and 11 white animals were genotyped.

**Results:**

Based on a list of colour genes of the International Federation of Pigment Cell Societies, a non-synonymous mutation with exchange of a glycine residue at position 291 of the tyrosinase protein by arginine was identified as the cause of dilution of the coat colour. A gene test led to exactly matching genotypes in all examined animals. The study showed that 14% of the brown animals carry the white gene. This provides a simple and reliable way of conservation for the white animals. However, results could not be transferred to another, unrelated red deer population with white animals. Although no brown animals with a white tyrosinase genotype were detected, the cause for the white colouring in this population was different.

**Conclusions:**

A gene test for the conservation of white red deer is available for the population of the Reinhardswald. While mutations in the tyrosinase are commonly associated with oculocutaneous albinism type 1, the amino acid exchange at position 291 was found to be associated with coat colour dilution in *Cervus elaphus*.

## Background

Genes associated with white coat colour and dilution were most extensively studied in mice [1]. At least 378 genes (171 cloned genes and 207 uncloned genes) involved in white colour or dilution are available from the International Federation of Pigment Cell Societies (a selection of genes, regulary involved in white coat colour is shown in Table 1). Their proteins are involved in melanocyte development and migration (Tyrosinase protein kinase KIT [KIT], Kit ligand [Kitlg], Endothelin 3 [Edn3], Endothelin receptor type b [Ednrb]), the biosynthesis of melanin (Tyrosinase [Tyr], Tyrosinase related protein 1 [Tyrp1], Dopachrome tautomerase [Dct]), the control of melanogenesis (Proopiomelanocortin 1 [Pomc1], Melanocortin 1 receptor [Mc1r], Agouti signalling peptide [Agouti], Microphthalmia-associated transcription factor [Mitf]), melanosome biogenesis (Silver [SILV], Pink-eyed dilution [P], Adaptor related protein complex 3 [Ap3]) and melanosome transport (Melanophilin [Mlph], Myosin-Va [Myo5a], Ras-related protein b27a [Rab27a]). TYR, TYRP1/gp75 and DCT/Tyrosinase related protein 2 [TYRP2] are involved in the biosynthesis of the different kinds of melanin [2]. TYR catalyses the rate-limiting reaction in melanin synthesis, converting tyrosine to dopaquinone and oxidizing 5,6-dihydroxyindole (DHI) to indole-5,6-quinone [3]. TYRP1 and DCT function more downstream in the melanin biosynthetic pathway [3, 4]. These processes are regulated by additional proteins like the Premelanosome protein 17 (Pmel17, gp100) [5], the Pink-eyed dilution protein (P) [6], and the Melanoma antigen recognized by T-cells protein (MART-1) [7]. The Mc1r and its substrate, the α-melanocyte stimulating hormone (α-MSH) are involved in modifications of coat colour [8]. Further factors involved in transcriptomic regulation are the MITF, and a basic-helix-loop-helix (bHLH) transcription factor [9]. In other ruminants, for example in cattle, at least 9 different genes have been associated with white colouring: ASIP [10], DCT [1], TYR [1, 11], TYRP1 [1], KIT [12], KITLG [13], MC1R [14], mast cell growth factor (MGF) [15], MITF [16] and PMEL [17].

**Table 1:** Selection of genes frequently associated with white coat colour

abbreviation	name	function	examples in ruminants	references
AGOUTI	Agouti gene	Control of melanogenesis		2
AP3	Adaptor protein 3	Melanosome biogenesis		2
ASIP	Agouti-signalling peptide	Control of melanogenesis	x	10
DCT	Dopachrome automerase	Melanin biosynthesis, different kinds of melanin	x	2,4
EDN3	Endothelin 3	Melanocyte development and migration		2
EDNRB	Endothelin B receptor	Melanocyte development and migration		2
KIT	KIT gene	Melanocyte development and migration	x	12
KITLG	Kit ligand	Melanocyte development and migration	x	13
MART-1	Melanoma antigen recognized by T-cells 1	Regulation of melanin synthesis		7
MC1R	Melanocortin 1 receptor	Control of melanogenesis		8,14
MGF	mast-cell growth factor	Control of melanogenesis	x	15
MITF	Microphthalmia-associated transcription factor	Control of melanogenesis	x	16
MITF	microphthalmia-associated transcription factor	Transcriptomic regulation		9
MLPH	Melanophilin	Melanosome transport		2
MYO5A	Myoxin, heavy polypeptide 12	Melanosome transport		2
P	Pink-eyed dilution gene	Melanosome biogenesis		6
PMEL17	premelanosome protein 17	Regulation of melanin synthesis	x	5,17
POMC1	Proopiomelanocortin 1	Control of melanogenesis		
Rab27a	RAS-related gene 27	Melanosome transport		2
SILV	Silver	Melanosome biogenesis		2
TYR	Tyrosinase	Melanin biosynthesis, different kinds of melanin	x	1,2,3,11
TYRP1	Tyrosinase-related protein 1	Melanin biosynthesis, different kinds of melanin	x	1,2,3

X: examples in ruminants available

In addition to colour inheritance in cattle [18], information is also available on sheep [19], goat [20] and buffalo [21]. However, nothing is known about colour inheritance in Cervids. Although so far only a few genes seem to be associated with the whitening of cattle, there is still a wide range of candidate genes to be considered in the search for the genetic cause of the whitening of red deer. White coat colour or dilution are extremely rare in red deer. In Germany there are two populations with white individuals, one in the Reinhardswald in the North of Hesse and one in Siegen-Wittgenstein in North Rhine-Westphalia. Within the approximately 1000 individuals of the red deer population of the Reinhardswald, about 50 white animals are suspected. Similar conditions exist in Siegen-Wittgenstein. It is important for the conservation of the white animals to identify the responsible gene variants and develop gene markers. This is the only way to make targeted statements about the distribution of the white gene variant in the population. However, up to now, nothing is known about the genes that are responsible for the white coat colour. The aim of the present work was therefore to first limit potential candidate genes by means of genome-wide single nucleotide polymorphism (SNP) analysis and then to identify the most highly white colour-associated SNPs.

## Results

Sequencing of the hind and her calf resulted in a coverage of 12.41 and 12.96 fold, respectively. Resulting reads were aligned to the reference sequence of the bovine genome (UMD 3.1). A total of 34.24 and 35.77 Gigabases mapped 82.18 and 82.08% of the whole genome, respectively. Comparing hind and calf, around 9.9 million SNPs were identified.

After variant calling and annotation, 8570 SNPs were extracted as a subset of SNPs based on a list of colour genes detected in mice, human and zebrafish (International Federation of Pigment Cell Societies). 2185 of them were non-synonymous (ns) and 6565 synonymous (s) SNPs that covered 115 genes. Among them were *ASIP* with 3 ns and 4 s SNPs, *DCT* with 19 ns and 35 s SNPs, endothelin receptor type b (EDNRB) gene with 6 ns and 14 s SNPs, KIT with 18 ns and 78 s SNPs, MC1R with 11 ns and 46 s SNPs, TYR with 17 ns and 43 s SNPs, and TYRP1 with 24 ns and 43 s SNPs. Synonymous SNPs were excluded from further processing. Following the hypothesis of a recessive inheritance of the white colour, we expected the genotype of the white hind to be homozygous for the white allele and the brown calf to be heterozygous. All genes and SNPs that did not correspond to this assumption were sorted out, resulting in 15 genes with 21 ns SNPs to be further examined (Table 2). For each of these SNPs a polymerase chain reaction (PCR) system was established to test the association of the gene variant with the phenotypes of a sample of white and brown individuals of the population (Table 2). The SNP at the TYR gene was the only one with a 100% match between genotype and phenotype.

**Table 2:** List of candidate genes after extracting non-synonymous colour genes and their association with coat colour in red deer populations from Reinhardswald and others. All figures originally referring to the bovine genome were recalculated so as to fit to the recently published genome sequence CerEla1.0 of *Cervus elaphus*

*Cervus elaphus*							not Reinhardswald		Reinhardswald		*Bos taurus*	
CEC	chr_pos	gene	prot_len	pos	aa variant	SNP	brown	white	brown	white	BTA	chr-pos
3	47,510,875	ADAMTS20	1915	1134	p.1134 V > I	0 = G,1 = A	GG,AG	GG,AA	AA,AG	AA,AG	5	37,227,378
3	47,511,824	ADAMTS20	1915	1204	p.1204I > T	0 = C,1 = T	CT, TT	CC, TT	CC,CT	CC,CT,TT	5	37,228,964
12	126,879,585	APC	2851	2720	p.2720 T > I	0 = G,1 = A	GG,AG	AA,AG	AA,AG,GG	AA,AG,GG	10	1,118,743
30	26,850,856	ATP7B	1505	604	p.604D > Y	0 = C,1 = A	AA,AC	AA	AA, AC	AA	12	21,442,340
5	124,958,667	DPH1	438	273	p.273Q > R	0 = A,1 = G	AG	GG,AG	GG,AG	AA,GG,AG	19	23,643,044
1	30,427,888	DRD2	445	323	p.323A > T	0 = C,1 = T	TC, TT	TC, TT, CC	CT,TT	CC,CT,TT	15	24,305,495
7	4,579,141	DST	5653	918	p.918A > V	0 = C,1 = T	TT,TC	CC,TT	CC,CT	CC,CT,TT	23	3,499,604
7	4,697,844	DST	5653	3005	p.3005 N > S	0 = A,1 = G	GG,AG	AA,GG	AA,AG	AA,AG	23	3,584,163
5	8,504,491	HPS4	681	64	p.64 V > I	0 = G,1 = A	GG,AG	AA, GG	AG,GG	GG	17	68,389,531
5	8,509,383	HPS4	681	143	p.143I > V	0 = T,1 = C	CC, CT, TT	CC, CT, TT	CT,TT	CT,TT	17	68,383,336
5	8,515,161	HPS4	681	298	p.298S > L	0 = G,1 = A	AG, GG	AA, GG	AG,GG	AG,GG	17	68,377,269
5	gap	HPS4	681	571	p.571A > V	0 = C,1 = T	CC, TC	CC, TT	CT,CC	CT,CC	17	68,372,291
23	26,036,482	ITGB1	798	670	p.670I > V	0 = A,1 = G	AG, GG	AG,GG	AG,GG	GG	13	20,282,477
3	35,496,676	KRT75	543	35	p.35A > T	0 = G,1 = A	GG, AG	AA,GG	AG,GG	AA,AG,GG	5	27,630,776
1	70,321,949	MYO7A	2293	1779	p.1779R > Q	0 = G,1 = A	GG,AG	AA,GG	AG,GG	AA,AG,GG	15	57,402,107
11	4,864,962	NOTCH1	2900	40	p.40 V > A	0 = A,1 = G	SNP not confirmed by Sanger sequencing				11	104,015,685
11	4,865,366	NOTCH1	2900	59	p.59P > R	0 = G,1 = C	CC,GC	CC	CC,CT,GG	CC,CT,GG	11	104,015,279
21	458,420	RECQL4	1218	600	p.600G > R	0 = G,1 = A	AA, AG	GG,AA	GG	GG	14	1,617,571
12	53,771,046	SLC24A5	501	177	p.177A > V	0 = G,1 = A	GG, AG	AA, AG	AG	AA	10	62,475,788
1	55,722,734	TUB	508	163	p.508A > V	0 = G,1 = A	GG,AG	AA,AG	AA,AG,GG	AA,AG	15	45,040,996
2	na	TYR	530	291	p.291G > R	0 = C,1 = T	CC, CT	TT,CC	CT, CC	TT	29	6,451,778

*CEC Cervus elaphus* chromosome, *chr_pos* position on the *Cervus elaphus* chromosome (in bp), *gene* name of the gene, *prot_len* length of the corresponding protein, *pos* position of the amino acid substitution in the protein, *aa variant* amino acid variant, *brown/white* genotype(s) of the brown and white animals, respectively; gap: gap in CerEla1.0, *na* not annotated. In a first step, all genes were tested with 3 brown and 3 white animals from the Reinhardswald and 3 brown and 3 white animals not from the Reinhardswald. TYR was tested with 194 brown and 11 white samples from the Reinhardswald and 21 brown and 9 white animals not from the Reinhardswald

The sequence of the five exons of the red deer tyrosinase mRNA, spanning 1593 bases showed a genetic similarity with the sequences of the human and bovine tyrosinase of 86 and 97%, respectively.

Sequencing of the hind and her calf with the reference genome CerEla 1.0 resulted in a coverage of 9.58 and 10.05 fold, respectively. A total of 32.36 and 33.94 Gigabases mapped 92.0 and 92.0% of the whole genome, respectively. Comparing hind and calf, around 11 million SNPs were identified.

The results were verified by sequencing the same two individuals using the later available genome sequence for *Cervus elaphus* (CerEla1.0). Nineteen of the 21 SNPs from 14 of the 15 candidate genes could be verified with CerEla1.0. One SNP in HPSA4 on *Cervus elaphus* chromosome (CEL) 5 and the SNP in the tyrosinase gene (CEL 2) could not be detected because of a gap in CerEla1.0 at heat shock protein family A (Hsp70) member 4 (HSPA4) and because the respective region of the tyrosinase gene was not yet annotated in CerEla1.0.

In the population of the Reinhardswald, there was no brown individual with genotype AA of TYR and none of the white phenotypes had genotype GG or GA. Thus, the inheritance of the white colour in the red deer of the Reinhardswald was established as autosomal recessive. The tyrosinase gene is located on *Cervus elaphus* chromosome (CEC) 2. The SNP c.871G > A in the tyrosinase gene is located within a highly conserved region and results in an amino acid substitution of Glycine by Arginine. From 194 brown red deer of the Reinhardswald 86% were homozygous and 14% were carriers of the white allele. Considering the estimation by the forest officers of the Reinhardswald of 50 white animals among the total population of around 1000 red deer (approximately 5%), genotype frequencies for GG, GA and AA were estimated as 81.7, 13.3 and 5%, respectively. Under this assumption, the allele frequencies are estimated to be 88.4% (G) and 11.6% (A), respectively. Thus, the estimated genotype frequencies deviate significantly from the Hardy-Weinberg equilibrium (*p* < 0.001). The expected values are 78.1% (GG), 20.5% (GA) and 1.3% (AA), respectively. There was no obvious phenotypic difference between carriers of the GG and the GA phenotype.

C.871G > A was not associated with red and brown coat colour in the unrelated German red deer population Siegen-Wittgenstein. However, the TYR-genotype AA was never detected in a brown individual regardless of its origin.

## Discussion

Because a *Cervus elaphus* reference genome was not available at the time of sequencing, sequence reads of the red deer were aligned to the reference sequence of the bovine genome (UMD 3.1). After CerEla1.0, the complete genome sequence of the red deer was published [22], the sequences of the hind and her calf were realigned to CerEla1.0 as the reference sequence. With the use of CerEla1.0 versus UMD 3.1, 92% instead of 82% of the genome of the hind and the calf could be mapped. At the same time, the number of SNPs between calf and mother increased by about 10%. As expected, sequencing based on *Cervus elaphus* sequences proved to be superior than sequencing on the basis of *Bos taurus* sequences.

However, since the TYR gene was not annotated in CerEla1.0 the responsible SNP for the white phenotype in the Reinhardswald red deer population had no chance to be detected. This is not unexpected, since 21,880 genes are annotated for the bovine genome in contrast to 19,368 for the genome of *Cervus elaphus*. Nevertheless, the high degree of agreement even of microsatellite sequences, between red deer and other ungulates, especially cattle [23, 24] justified the use of the bovine genome as reference sequence. Indeed, red deer sequences homologous to 82% of the bovine genome were mapped, including 9.9*10^6^ SNPs. We were confident that the coding sequence ranges in particular would show a good match between red deer and bovine genome. In fact, 8570 SNPs were extracted after variant calling as a subset based on a list of colour genes (International Federation of Pigment Cell Societies). Twenty one SNPs in 15 candidate genes corresponded exactly to the requirements of a homozygous white hind and its heterozygous brown calf. However, only one SNP, located in the TYR gene matched exactly to the total sample with 194 brown and 11 white animals of the Reinhardswald population. The probability of a random match between genotype and phenotype (0.5^205^) in this number of animals corresponds to 1.94*10^− 62^. Although the exact number of white individuals is not known, the responsible forestry authority assumes about 50 white animals, within a total population of about 1000 red deer. Using the prevalence of heterozygous brown red deer, this results in a significant deviation from the Hardy-Weinberg equilibrium with too high a proportion of homozygous white genotypes. This could be explained by the fact that no white red deer had ever been shot up to the time of the study (selection). The mixed-bred, brown animals, on the other hand, were hunted without any difference to the clean-brown red deer. Factors that could have led to the preferred reduction of white individuals, such as predators (e.g. wolf or lynx), were not present in the region studied. The selection for white red deer results in particular from the fact that the reference to its existence is used as a unique selling point and tourist advertising object for the region. In this context, citizens’ initiatives have repeatedly been campaigning for the preservation of white individuals.

Since white animals were also sporadically the victims of traffic accidents, it was an important question to investigate whether the 50 estimated individuals were left to their own or whether they could be regarded as an integrated part of the total population. The present study showed with the proof of the heterozygous brown individuals that the white allele is deeply anchored in the population and that statistically, one to two new white calves can be expected from the mating of heterozygous brown animals per year.

Tyrosinase is the key enzyme in the synthesis of melanin. It catalyses the rate-limiting step, the hydroxylation of the amino acid tyrosine to dopaquinone [25] and subsequently the oxidation of 5,6-dihydroxyindole (DHI) to indole-5,6-quinone [3]. Hundreds of mutations in the tyrosinase gene including missense, nonsense, frameshift, splice site mutations and a deletion of the entire coding sequence have been identified and associated with oculocutaneous albinism type I (OCA1 [26]; http://www.ifpcs.org/albinism/). This is an autosomal recessive disorder, associated in most cases with severe hypopigmentation of the skin, hair and eyes, most often accompanied by nystagmus, foveal hypoplasia and reduced visual acuity [26]. Only few polymorphisms in the coding region of the gene have been described [27]. Besides human and mouse, TYR mutations associated with albinism have been found in rabbits [28], cats [29], rats [30], ferrets [31], minks [32], donkeys [33], humpback whale [34] and cattle [11].

In addition to the extensive cases of albinism, mutations in mice have also been described in connection with coat dilution, particularly in connection with pheomelanin [35–37]. However, pheomelanin coat colour dilution in French cattle breeds could not be correlated with tyrosinase [1]. Colour variants of the Bactrian camel [38] and dilution in the coat colour of Alpaca [39] could not be associated with mutations in the *TYR* gene.

White deer is found only sporadically. We only know of one single reference that deals with microsatellite analysis for the control of inbreeding and genetic diversity in a population of white red deer in Czech Republic [40]. The causes for the colour of the white coat in this species are completely unknown. The coat colour of the white individuals is diluted, but they are not albinos. The eyes are pigmented. The polymorphism that is responsible for the dilution led to an amino acid exchange at position 291, where the amino acid glycine is found in humans, cattle and red deer. Mutations in humans are not known. Amino acid 291 lies outside known functional areas of the tyrosinase protein. In animals with a white coat, glycine was replaced by arginine. Arginine is basic, positively charged and hydrophilic. Glycine is an uncharged, apolar and hydrophobic amino acid. Although PANTHER14.1 (http://pantherdb.org/tools/csnpScoreForm.jsp) predicted this amino acid exchange as benign, this chemical difference may alter the efficacy of tyrosinase without a complete failure. Vitkup et al. [41] and Khan and Vihinen [42] concluded that mutations at arginine and glycine residues are together responsible for about 25 to 30% of genetic diseases. The same mutation has been described in a white Korean Hanwoo cattle (gene bank AccNo YQ513971). Unfortunately, a detailed phenotype of the cattle is not available. Thus it is not clear, whether the cattle suffer from complete OCA1 or just a dilution of the coat colour.

The extension of the study to a second, unrelated red deer population revealed no brown carriers of the AA variant; however, white animals without the AA genotype at position 291 of the tyrosinase protein were found. This indicates that in this population (Siegerland-Wittgenstein) another, unknown gene variant segregates, which leads to dilution of the coat colour. Thus, although the tyrosinase mutation is responsible for the white colouring of the deer of the Reinhardswald, other previously unknown mutations are to be expected in other populations of white red deer.

In addition to the result of anchoring the white individuals of the Reinhardswald in the brown red deer population, the study can also serve to document dispersal paths and migration movements to neighbouring red deer areas and to distinguish red deer populations with white individuals from each other. For this purpose, more red deer populations need to be tested for the presence of the c.871G > A tyrosinase gene variant. The gene test can also be used to investigate the influence of the tyrosinase gene variant on physical development, fertility and adaptability within the segregating population. It is anecdotically assumed that the white deer of the Reinhardswald were imported from southeastern Europe in the sixteenth century, scattered throughout the region in the turmoil of the Thirty Years’ War in Europe and have survived to this day. By screening different Southeast European red deer populations, it could be possible to decipher the origin of the white red deer of the Reinhardswald in future studies.

Furthermore, the results show an enormous potential for the use of well-established reference genomes of closely related species for genomic analyses (especially at gene level) in species for which no reference genome is yet available.

## Conclusion

The identification of the gene variant responsible for the white coloration and the quantification of heterozygous animals provided evidence that the few white animals are not an independent population. Rather, the white allele is widespread throughout the entire population via the heterozygous, brown animals.

## Materials and methods

### Red deer population

The Reinhardswald is a part of the Weserbergland, one of the biggest coherent forest areas of Germany and is located in the North of the Federal State of Hesse (51° 30′ N, 9° 34’O). The forest covers an area of 183 km^2^ and, according to the Reinhardswald red deer association, has a census size of about 1000 animals of which about 50 animals are white.

### The phenotype

**Fig. 1:**
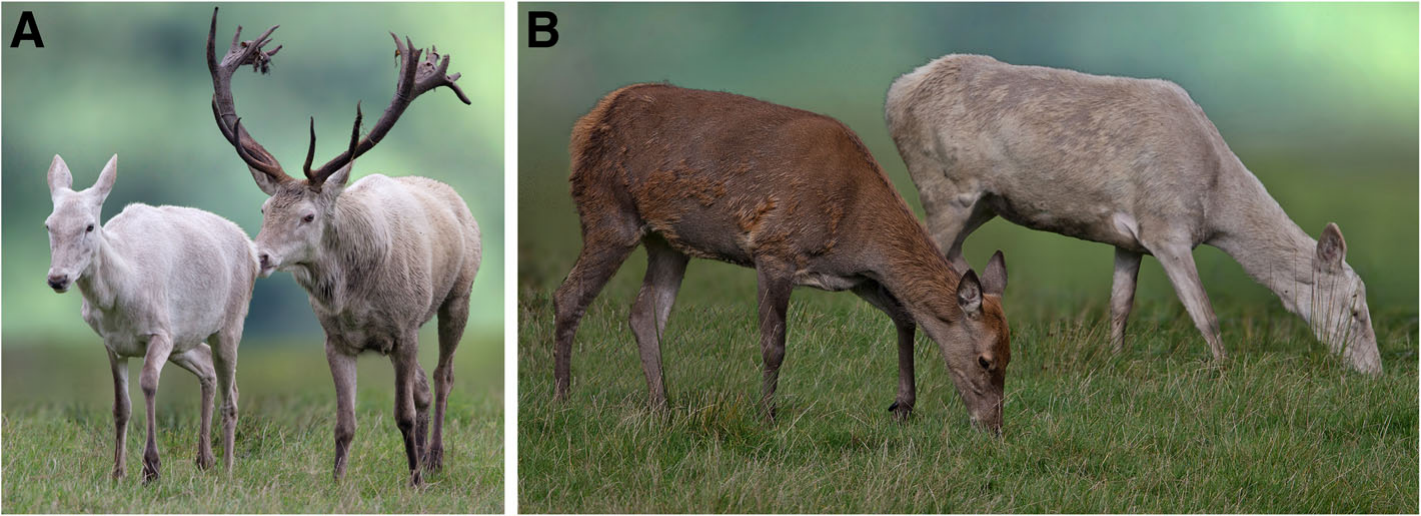
The hind shows a slightly stronger brightening than the stag. The eyes are clearly pigmented with both animals (**a**). Comparison between a normal brown hind and a hind with white coat colour (**b**)

The white deer of the Reinhardswald are not albinos. The coat colour is very pale, stronger in summer than in winter. The dilution is qualitatively distinguishable by eye. Eyes and claws are normally pigmented or slightly lightened. Apart from the coat and eye colour, the white animals do not differ from the brown of the population in height, weight and habitus (Fig. 1). However, there is no detailed information on the phenotype (histology, physiology, biochemistry) available.

### Sample collection

During hunting seasons 2013 to 2015 tissue samples from brown (*n* = 194) and white (*n* = 3) red deer and samples from antlers of white red deer (*n* = 8) were collected. For sequencing, samples of two female animals (one white adult hind with its brown calf) were available. Samples were taken from existing antlers and frozen tissue samples provided by those authorised to practise hunting. No animals were killed specifically for the study. No live animals were sampled and no dropping antlers were sought or collected for the study. All samples were accompanied with information about age, weight, colour, and hunting ground. In addition, the presence/absence of white animals in the deer pack from which a sample was taken was recorded.

Further samples from brown (*n* = 21) and white (*n* = 9) red deer were collected in exactly the same way in Siegen-Wittgenstein, another area with brown and white animals. Reinhardswald and Siegen-Wittgenstein are separated by 110 km, a fenced motorway, several country roads and a red deer-free area. Both populations were not related or linked with each other as shown by a population differentiation test implemented in Genepop (see below).

Samples from antlers were taken as drill core samples from the base and stored dry at ambient temperature. Tissue samples were frozen at − 20 °C until use.

### DNA extraction

Genomic DNA was extracted from tissue samples and antler drill cores with the Instant Virus RNA Kit (Analytik Jena, Germany). This kit was thoroughly tested against DNA extraction kits and its ease of use and its efficiency in extracting DNA was found to be comparable or even superior. Antler drill cores (0.1 to 0.3 g) were treated in a beadmill (MM200, Retsch, Germany) at a frequency of 25 Hz for 2 min. Tissue samples were suspended in 450 μl of lysis buffer and subsequently treated in the same way as the antler drill cores. All following steps were performed according to the manufacturer’s instructions. The extracted DNA was eluted with 60 μl of RNAse-free water.

DNA concentration was measured photometrically with the Nanodrop 2000 spectrophotometer (Thermofisher, USA) and the Qubit 2 system (Qubit dsDNA br assay kit and Qubit dsDNA hs assay kit, Thermofisher, USA).

### DNA quality control and next generation sequencing

The DNA of the hind and calf were provided for genomic sequencing. The amount of DNA was quantified through qPCR with the Kapa Library Quantification Kit (Kapabiosystems, USA) and diluted to 20–30 ng/μl for library preparation (TruSeq DNA PCR-free sample preparation Kit, Illumina, USA). Fragment sizes of the libraries were visualized with a BioAnalyzer 2100 (Agilent Genomics, USA).

Quality controlled libraries were sequenced using the HiSeq 2500 instrument (Illumina, USA). Paired-end libraries (2 × 126 bp reads) were sequenced with a mean coverage of ten times.

Prior to further processing raw data were quality checked for overrepresented and duplicate sequences with FastQC (http://www.bioinformatics.babraham.ac.uk/projects/fastqc/).

Raw sequences were then converted from a base call file (bcl) to fastq files and mixed probes were demultiplexed through the program bcl2fastq Conversion Software from Illumina (http://emea.support.illumina.com/downloads/bcl2fastq_conversion_software_184.html?langsel=/de/). Because a *Cervus elaphus* reference genome was not available at the beginning of the study, resulting reads were at first aligned to the reference sequence of the bovine genome (UMD 3.1 [43]) and in a second step to the *Cervus elaphus* reference sequence CerEla1.0, both using the *BWA-MEM* algorithm (https://arxiv.org/abs/1303.3997). After processing of data, single files were merged and converted from the *SAM* to the *BAM* format with *SAMtools* [44]. Duplicated reads were marked by the *PICARDtools* command *MarkDuplicates* (https://github.com/broadinstitute/picard/).

### Variant calling, annotation and identification of candidate variants

To identify single nucleotide polymorphisms (SNPs) as well as short insertion and deletion polymorphisms (INDELs) in the annotated reads of the two sequenced red deer samples, we used the *mpileup* algorhithm implemented in *SAMtools* [44]. With the *filter* algorithm from *PICARDtools* (https://github.com/broadinstitute/picard/) called variants were filtered by excluding all SNPs within 3 basepairs of an INDEL and with lower *QUAL* score, and by excluding INDELs within 2 basepairs of another INDEL.

For the functional annotation of each called SNP we adapted the *VariantEffectPredictor (VEP)* from Ensemble [45].

Furthermore, we extracted a subset of SNPs based on a list of colour genes detected in mice, human and zebrafish (International Federation of Pigment Cell Societies; http://www.ifpcs.org/albinism/). Resulting *VEP* annotated files containing only genomic regions coding for coat colour were checked on the basis of a recessive genetic inheritance model for non-synonymous impacts of the mutations.

### Validation of candidate SNPs

SNPs were selected in a hierarchical procedure as candidate SNPs for further processing. First and foremost, they had to be within the range of colour genes specified by the International Federation of Pigment Cell Societies. The second prerequisite was that the SNP was non-synonymous. The SNP had to be homozygous for the hind and heterozygous for the calf. The responding 21 candidate-SNPs (15 different genes) were validated by Sanger sequencing (ABI 3500 genomic analyzer). For this purpose, regions including the candidate SNPs were PCR amplified and sequenced. PCR primers were designed (https://primer3plus.com/cgi-bin/dev/primer3plus.cgi) from the NGS data in combination with data from the *Bos taurus* reference genome (UMD 3.1). Later the SNPs were verified with CerEla1.0, the *Cervus elaphus* reference genome.

### Pyrosequencing

Genotypes of animals were detected by pyrosequencing on a Pyromark Q96 ID system (Qiagen, Germany) and sequences were analyzed with the Pyro-Mark ID 1.0 Software (Qiagen, Germany).

PCR was performed in a total volume of 40 μl consisting of 20 μl of Multiplex Mastermix (Qiagen, Germany), 4 μl of primer mix (HW-TYRF 5′-TTTCCAGGATTGCGCAGTA-3‘, HW-TYRR 5‘-TGCAGCAGATTGGAGGAGTAC-3‘) with a final concentration of 0.4 μM, 12 μl of water and 4 μl of template DNA. Cycling conditions were as follows: initial activation of the DNA polymerase for 15 min at 95°C, followed by 35 cycles of denaturation at 94°C for 30 seconds, annealing at 52°C for 90 seconds and extension at 72°C for 30 seconds, followed by final extension at 72°C for 10 min. Quality and amount of PCR products was checked by electrophoresis on 1.5% agarose gels stained with Midori Green Advance (Biozym, Germany). PCR products immobilized to streptavidin-sepharose beads were released in 40 μl of 5 μM sequencing primer (HW-TYRS 5’-ATGGTCCCTCAGACG-3′) and subjected to pyrosequencing.

### Population genetic analysis

To test the effect of the white gene in another population red deer from Siegen-Wittgenstein (21 brown and 9 white animals) were included. Phenotypically, no differences could be found between red deer originating from Reinhardswald and Siegen-Wittgenstein. Population genetic analysis using microsatellites [46] was conducted to verify the independence of the two populations. The population differentiation test [47] implemented in Genepop (https://kimura.univ-montp2.fr/~rousset/Genepop.htm) was performed as an exact G test with the following Markov chain parameters: dememorisation length of 100,000 and 100 batches with 10,000 iterations per batch.

## Data Availability

Data and materials are available from the authors on reasonable request. The tyrosinase sequence and the polymorphism are available in the NIH genetic sequence database GenBank® (Accession number MN913379; https://www.ncbi.nlm.nih.gov/nucleotide/MN913379).

## Abbreviations

°C: Grades Celsius
A: Adenine
AA: Homozygous for Adenine
AccNo: Accession number
AG: Heterozygous for Adenine and Guanine
Agouti: Gene of the Agouti signalling peptide
Ap3: protein of the Adaptor related protein complex 3
ASIP: Agouti signalling peptide
bHLH: basic-helix-loop-helix
bp: basepairs
c.871G > A: polymorphism of the coding sequence at position 871 with exchange between adenine and guanine
CEL: *Cervus elaphus* chromosome
CerEla 1.0: Name of the reference genome from *Cervus elaphus*
DCT: Dopachrome tautomerase gene
Dct: Dopachrome tautomerase protein
DHI: 5,6-Dihydroxyindole
DNA: Desoxyribonucleic acid
Edn3: Endothelin 3 protein
EDNRB: Endothelin receptor type b gene
Ednrb: Endothelin receptor type b protein
g: Gramm
G: Guanine
GG: Homozygous for guanine
gp100: glycoprotein 100
HPSA4: Heat shock protein family A (Hsp70) member 4 (HSPA4)
HZ: Herz (1/s)
INDEL: Insertion-deletion mutation
Kit: Protein of the Tyrosine-protein kinase KIT
KIT: Tyrosine-protein kinase KIT gene
KITLG: Kit ligand (gene)
Kitlg: Ligand of the Kit (protein)
Km: kilometer
MART-1: Melanoma antigen recognized by T-cells gene
MC1R: Melanocortin 1 receptor gene
Mc1r: Melanocortin 1 receptor protein
MGF: Mast cell growth factor
min: minutes
MITF: Microphthalmia-associated transcription factor gene
Mitf: Microphthalmia-associated transcription factor protein
Mlph: Melanophilin
mRNA: messenger ribonucleic acid
Myo5a: Myosin-Va (protein)
N: Number
ng: nano Gramm
ns: non-synonymous
OCA1: Oculocutaneous albinism type 1
P: Pink eyed dilution
PCR: Polymerase chain reaction
PMEL: Gene of the Premelanome protein
Pmel17: Premelanosome protein 17
Pomc1: Proopiomelanocortin 1 protein
qPCR: quantitative PCR
Rab27a: Ras-related protein b27a
s: synonymous
SILV: Silver (gene)
TYR: Tyrosinase gene
Tyr: Tyrosinase protein
Tyrp1: Tyrosinase related protein-1 (protein)
TYRP1: Tyrosinase related protein-1 gene
TYRP2: Tyrosinase related protein-2 gene
UMD 3.1: Name of the bovine reference genome sequence used
α-MSH: α-melanocyte stimulating hormone
μl: microliter
μM: micromolar
